# Respiratory Manifestations and Their Physical, Psychological, and Social Impacts in Ehlers-Danlos Syndromes and Generalized Hypermobility Spectrum Disorders: A Narrative Review

**DOI:** 10.3390/jcm14124126

**Published:** 2025-06-11

**Authors:** Noor Al Kaabi, Encarna Camacho, Ani Orchanian-Cheff, Vanessa Silano, Laura McGillis, Wing Ting Truong, W. Darlene Reid, Chung-Wai Chow, Clodagh M. Ryan, Maxwell Slepian, Daniel Santa Mina, Hance Clarke, Nimish Mittal, Dmitry Rozenberg

**Affiliations:** 1 Division of Respirology, University Health Network, Toronto, ON M5G 2C4, Canada; noor.alkaabi@utoronto.ca (N.A.K.); camacho.encarna@outlook.com (E.C.); vanessa.silano@uhn.ca (V.S.); chung-wai.chow@uhn.ca (C.-W.C.);; 2 Temerty Faculty of Medicine, University of Toronto, Toronto, ON M5R 0A3, Canada; hance.clarke@uhn.ca (H.C.); nimish.mittal@uhn.ca (N.M.); 3 GoodHope Ehlers-Danlos Syndrome Program, University Health Network, Toronto, ON M5G 2C4, Canada; laura.mcgillis@uhn.ca (L.M.); wingting.truong@uhn.ca (W.T.T.); maxwell.slepian@uhn.ca (M.S.); daniel.santamina@uhn.ca (D.S.M.); 4 Library and Information Services, University Health Network, Toronto, ON M5G 2C4, Canada; ani.orchanian-cheff@uhn.ca; 5 KITE—Toronto Rehab, University Health Network, Toronto, ON M5G 2C4, Canada; darlene.reid@utoronto.ca; 6 Department of Physical Therapy, University of Toronto, Toronto, ON M5G 1V7, Canada; 7 Interdepartmental Division of Critical Care Medicine, University of Toronto, Toronto, ON M5R 0A3, Canada; 8 Faculty of Kinesiology and Physical Education, University of Toronto, Toronto, ON M5R 0A3, Canada; 9 Department of Anesthesia and Pain Management, University Health Network, Toronto, ON M5R 0A3, Canada

**Keywords:** signs and symptoms, respiratory, Ehlers-Danlos syndromes, health-related quality of life, patient-reported outcome measures

## Abstract

Ehlers-Danlos Syndromes (EDS) and Generalized Hypermobility Spectrum disorders (G-HSD) are a group of genetic, connective multi-systemic disorders that can affect the musculoskeletal, gastrointestinal, cardiovascular, and respiratory systems. Respiratory manifestations in EDS/G-HSD can contribute to decrements in health-related quality of life (HRQL); however, these relationships have not been previously characterized. We aimed to review the association of respiratory manifestations with the physical, psychological, and social domains of HRQL in EDS/G-HSD. A comprehensive search was conducted using Ovid Medline, Embase, and CINAHL with the following terms: “Ehlers-Danlos Syndrome”, “Hypermobility Spectrum Disorders”, and “Quality of Life”. Selected studies in English that investigated the relationship between respiratory manifestations and HRQL domains in EDS/G-HSD were included in this narrative review from inception to March 2024. Twelve studies described the physical, psychological, or social domains of HRQL relating to respiratory manifestations. Dyspnea, wheezing, and expiratory flow limitations were associated with limitations in physical function and exercise intolerance. Respiratory manifestations were associated with increased fatigue, pain, anxiety, kinesiophobia, and deconditioning. This review highlights the consequences that respiratory manifestations have on the physical domains of HRQL, through limitations on physical activity and exercise. Future studies should aim to identify the impact that respiratory manifestations have on the psychosocial domains of HRQL and develop disease-specific patient-reported measures to evaluate these relationships.

## 1. Introduction

Ehlers-Danlos Syndromes (EDS) and Generalized Hypermobility Spectrum disorders (G-HSD) are genetic connective tissue disorders with 14 subtypes, characterized by multi-system manifestations, with a prevalence of 1:5000 [1,2,3,4]. The abnormal collagen and protein synthesis in EDS/G-HSD affects the structure and function of connective tissue [5]. Consequently, common systemic manifestations are seen in the neurological, gastrointestinal, cardiovascular, and respiratory domains, which impact daily function [6]. Pain and fatigue are the most common symptoms contributing to daily functional limitations and can range from mild to severe [7,8]. Anxiety, kinesiophobia, and depression are commonly reported psychological sequelae in the EDS/G-HSD population [9,10]. Collectively, these conditions comprise a range of debilitating symptoms that contribute to high disease burden and a lower health-related quality of life (HRQL) [6,11].

Recently, two narrative reviews summarized respiratory symptoms in EDS/G-HSD [12,13]. A framework by Bascom et al. highlights that respiratory symptoms, such as dyspnea and cough, may arise due to interactions between various structural, functional, and inflammatory processes [12]. Other significant respiratory manifestations were highlighted in a review by Chohan et al. that included asthma, chest tightness, and respiratory muscle weakness [13]. The literature highlights that individuals with EDS/G-HSD have been observed to have a 2- to 3-fold increase in respiratory symptoms such as dyspnea, cough, sputum or wheezing compared to healthy age- and sex-matched individuals [14]. Another contributing factor such as autonomic dysfunction has been associated with dyspnea in EDS/G-HSD [15,16]. Specifically, postural orthostatic tachycardia syndrome (POTS) is the most common form of dysautonomia in EDS/G-HSD with a prevalence of 18%, with associated symptoms of chest pain, heart palpitations, and dyspnea [17]. Additionally, there is an increased prevalence of atopic conditions in EDS/G-HSD such as mast cell activation syndrome (MCAS). MCAS is characterized by the abnormal release of histamine from mast cells resulting in inflammation and is a potential contributor of dyspnea and asthma [18,19]. Thus, a number of structural, functional, and inflammatory processes play an important role in mediating respiratory symptoms, which can be variable and overlapping.

In EDS/G-HSD populations, increased dyspnea, cough, and exercise intolerance have been associated with physical inactivity [20,21]. Increased dyspnea severity has also been associated with psycho-social factors such as anxiety, daily functional limitations, and work productivity in chronic lung diseases [22,23]. Previous literature [12,13] has summarized the characteristics of respiratory manifestations in tabular format in EDS/G-HSD [13]; however, no research studies to date have assessed the impact of these manifestations on HRQL. The respiratory symptoms that contribute to reduced HRQL are multifactorial in individuals with EDS and G-HSD and can be conceptualized in terms of three major health domains: (1) physical, (2) psychological, and (3) social. Given the unique and multi-faceted nature of EDS/G-HSD, it is important to consider the intersection of these domains that comprise HRQL.

In this review, we aimed to examine the impact of respiratory manifestations on HRQL in EDS/G-HSD. We describe these relationships using the physical, psychological, and social HRQL domains framework [24,25], summarizing the current literature and identifying areas for future research. The findings from this review will be helpful for patients, researchers, and health care providers in guiding health care management and studying clinical outcomes in the EDS/G-HSD population [26].

## 2. Materials and Methods

### 2.1. Literature Search

Given the limited knowledge in the area of respiratory manifestations and HRQL in EDS/G-HSD, we chose a narrative review to explore the evidence in this area. We searched Ovid Medline, Ovid Embase, and CINAHL Complete (EBSCOhost) on 10 January 2022 and subsequently updated on 14 March 2024. A search strategy was initially developed for Ovid Medline for the concepts of Ehlers-Danlos Syndrome and quality of life, with a combination of subject headings and keywords, as shown in Table 1**.** The search strategy was then translated to other databases (Supplementary Materials). We limited our references to English with no date limits. Studies were included on the basis of relevance to the relationship between respiratory symptoms and the domains of HRQL. Data extraction was performed for each selected study to include study population and design, inclusion and exclusion criteria, measurement instruments, outcomes and key findings.

In this review, the term respiratory manifestations refers to two categories: (1) respiratory symptoms including dyspnea, cough, wheezing, chest tightness, and thoracic pain; and (2) conditions associated with respiratory symptoms including obstructive sleep apnea (OSA), respiratory muscle weakness, and systemic conditions associated with dyspnea such as MCAS or dysautonomia. These respiratory manifestations have been shown to have significant effects on HRQL in EDS/G-HSD, which is in keeping with observations on HRQL across several chronic disease states [27,28,29,30].

### 2.2. Health-Related Quality of Life: Physical, Psychological, and Social Guiding Framework

**Figure 1:**
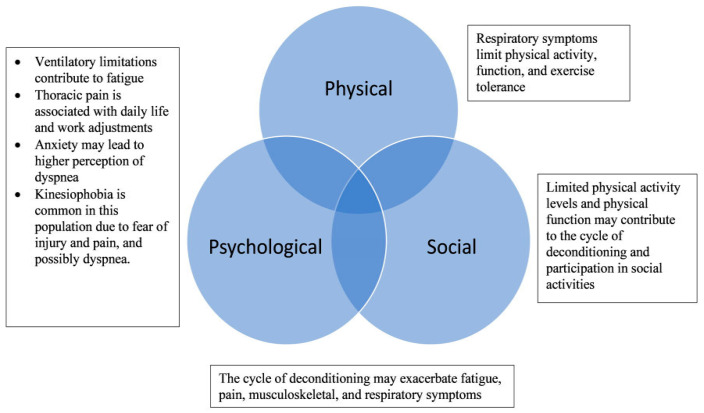
Physical, psychological, and social impact of respiratory manifestations in EDS/G-HSD. Conceptual diagram of the biopsychosocial model of health as proposed by Engel [32].

Respiratory symptoms have been known to affect individuals across various domains of health and well-being [13,31]. The physical, psychological, and social guiding framework stems from the biopsychosocial model of health which considers the interaction of multiple domains in contributing to health and well-being, and has been previously utilized to describe the overall impact on daily function in EDS/G-HSD [25,32]. Notably, all domains of HRQL have been observed to be lower in EDS/G-HSD in comparison to the general population and other chronic conditions [33,34]. Figure 1 provides a conceptual model for these relationships.

## 3. Results

### 3.1. Identified Articles

This narrative review is comprised of 12 selected studies on the basis of the relationship between respiratory manifestations and physical, psychological, and social domains of HRQL. Of the studies included, six were cross-sectional, two randomized controlled trials, three qualitative studies, and one retrospective chart review. The majority of study participants were female (87%) with mean or median age range being 18 to 67 years. Nine (75%) of the studies included physical domains, seven (58%) studies reported on psychological domains, and six (50%) reported on the social aspects of HRQL in EDS/G-HSD populations. Table 2 provides a summary of the key studies in this narrative review and their impact on HRQL. The studies collectively highlight the significant impact of respiratory symptoms on individuals with EDS/G-HSD.

Respiratory symptoms were assessed using self-reported questionnaires such as the St. George’s Respiratory questionnaire or the Nijmegen questionnaire. Pulmonary function tests such as spirometry and sniff nasal inspiratory pressure were used for evaluation of dyspnea and respiratory muscle strength. Most studies (*n* = 9) were focused on assessing the physical domains of HRQL, and outcomes included exercise capacity and physical activity levels. The biopsychosocial impact of EDS/G-HSD on daily life, including social participation and mental well-being, was described using qualitative thematic descriptions. The functional limitations, the associated psychological impact of disease, and overall implications on daily life were explored. Physical and psychological manifestations of EDS/G-HSD were reported to disrupt professional life and social functioning. Table 2 provides a summary of the studies in this review that were described to have an association with HRQL.

### 3.2. Physical Domains of Health-Related Quality of Life (HRQL): Physical Functioning, Activity, and Exercise Tolerance

Participation in daily, social, work or school activities commonly falls under the physical functioning domain of HRQL. In a study by Rombaut et al., 32 hEDS patients completed the Baecke questionnaire, a measure of habitual physical activity levels ranging from 3 to 15, with higher scores representing greater physical activity levels. EDS patients reported lower Baecke physical activity median scores [4.50 (range from 2 to 8)] compared to age- and sex-matched healthy controls [5.30 (range from 3 to 9, *p* = 0.027] [34,44]. Compared to healthy age- and sex-matched controls, individuals with EDS/G-HSD also had limitations in lower extremity muscle strength, endurance, and functional capacity [44,45].

Dyspnea, asthma, and atopic symptoms may be important contributors to physical limitations in the EDS/G-HSD population. In a study of 20 individuals with classical and vascular EDS, the majority of participants described experiencing mild dyspnea, with 70% reporting Medical Research Council (MRC) grade 1, whereas 15% experienced grade 2 dyspnea, and 10% having moderate dyspnea (grade 3) [46]. Morgan et al. observed increased cough, wheezing, and exertional dyspnea in 288 individuals with EDS/G-HSD [42], which were associated with limitations in exercise tolerance. Mechanical ventilatory constraints such as dynamic hyperinflation may also be associated with reduced exercise capacity in EDS/G-HSD, as shown in 12 hEDS participants undergoing constant load cycling [36].

Cardiac dysautonomia has also been associated with exercise intolerance and physical inactivity [38]. A retrospective evaluation of physical activity levels between dysautonomic and non-dysautonomic hEDS/G-HSD groups demonstrated that dysautonomia associated symptoms, such as dyspnea (36% prevalence), chest pain or tightness (34%) were associated with exercise intolerance [38]. Individuals with dysautonomia had a greater decrease in their exercise levels from moderate/vigorous exercise to light/no exercise (56% to 15%) after an exacerbation of their musculoskeletal symptoms compared to the non-dysautonomic group (68% to 40%) [38]. This highlights the considerable impact that comorbid disorders such as dysautonomia and its associated symptoms have on exercise intolerance. In accordance with the biopsychosocial model, patients with EDS experience challenges that extend beyond physical symptoms [12,47]. Limitations in their ability to participate in daily activities and exercise affect feelings of self-worth, frustration, and anxiety, which may contribute to interpersonal problems and reduced HRQL [47].

Respiratory muscle weakness has also been shown to accentuate symptoms of dyspnea and fatigue and contribute to limitations in exercise capacity in hEDS patients [48]. Reychler et al. observed that 20 participants enrolled in an inspiratory muscle training (IMT) program had reduced respiratory muscle strength with the mean maximal sniff nasal inspiratory pressure (65 ± 30% predicted). The 6-week IMT group had a 13% improvement in exercise capacity and 10% increase in forced expiratory volume in one second in comparison to the non-IMT group.

### 3.3. Physical Health-Related Quality of Life (HRQL): Pain and Fatigue

Given the multi-systemic nature of EDS/G-HSD, respiratory symptoms may exacerbate systemic symptoms and contribute to high “symptom burden”. The term “high symptom burden” has been used to capture the frequency and severity of symptoms, and their perceived impact on individuals [40]. One study observed that EDS patients with high symptom burden reported the highest number of respiratory symptoms, including dyspnea, wheezing, nasal congestion, cough, and chest pain [40]. This highly symptomatic group reported lower HRQL and greater pain severity, fatigue levels, and sleep disturbances compared to the other EDS groups with fewer respiratory symptoms.

Pain and fatigue are multidimensional symptoms [41,49,50,51,52]. Pain is a cardinal symptom in over 90% of individuals with EDS/G-HSD and has been recognized as one of the most frequent and disabling symptoms in this population [51]. Bodily pain is often reported effecting the lower limbs [51], as unstable and painful limb joints can often limit activities such as walking or instrumental activities of daily living that require bending. Upper airway symptoms and thoracic pain have also been reported in several case studies, which may further limit daily activities [53,54,55]. One study observed that 75% of singers diagnosed with EDS/G-HSD experienced throat pain, functional voice difficulties (e.g., hoarse voice), and other laryngeal difficulties (i.e., coughing, choking) [35]. These symptoms often necessitate several lifestyle adjustments, including the use of speech therapy, devices, sign language or lip reading to enhance communication [35].

More than 80% of EDS/G-HSD individuals report experiencing severe chronic fatigue that limits their daily and social activities [41,49,56]. Fatigue has been described as affecting social participation, well-being, and physical activity. Thus, fatigue is a key contributor to reduced HRQL in individuals with EDS/G-HSD [2,56]. Particularly, impairments have been observed in the physical function, social, and pain domains of the Short Form-36 (SF-36) [8]. EDS/G-HSD patients experiencing severe fatigue have lower SF-36 physical functioning scores in comparison to those with non-severe fatigue (37 ± 23 vs. 61 ± 27, *p* < 0.001). Those with severe fatigue also experience higher levels of psychological distress and functional impairment in daily activities [8].

Obstructive sleep apnea (OSA) has been shown to have negative effects on HRQL and fatigue in EDS/G-HSD. In a systematic review of 13 OSA studies, the authors observed an estimated OSA prevalence of 39% in individuals with joint hypermobility syndrome [57]. In a study of 100 EDS patients, excessive daytime sleepiness measured with the Epworth Sleepiness Scale, was associated with worse HRQL in comparison to healthy age- and sex-matched controls [58]. Lower scores were also noted across all domains of the SF-36 including physical, social, and mental health [58]. The working theory is that OSA disrupts sleep patterns and leads to higher levels of fatigue and daytime sleepiness, which can affect daily function, work and social activities, which are key determinants of HRQL [59].

### 3.4. Psychological Health-Related Quality of Life (HRQL): Anxiety and Kinesiophobia

Anxiety is commonly reported in individuals with EDS/G-HSD with an estimated prevalence of 60–75% [60]. High anxiety levels among EDS patients are likely due to associated comorbidities that can exacerbate mental health conditions, increase fear of injury, and perpetuate physical and social restrictions experienced by individuals with EDS and G-HSD [10]. Furthermore, about half of EDS patients with anxiety describe exertional dyspnea [61]. Individuals with hEDS with more severe functional respiratory complaints (e.g., dyspnea, chest pain, and chest tightness) scored higher on questionnaires evaluating depression, anxiety, and central sensitization syndromes (e.g., fibromyalgia), indicating greater severity of these symptoms [31]. Higher levels of anxiety may lead to increased sensitization to bodily sensations and awareness, thus worsening patient experiences and tolerance of pain in EDS/G-HSD [31,62].

Moreover, pain, fatigue, kinesiophobia, and other musculoskeletal limitations are well documented barriers to physical activity and exercise in individuals with EDS/G-HSD [39,51]. Qualitative studies describe that EDS/G-HSD patients often limit daily and social activities due to fear of musculoskeletal injuries [63]. Individuals with EDS/G-HSD often report high levels of kinesiophobia, or fear of movement due to concerns related to injury or aggravation of their pain [64].

### 3.5. Social Health-Related Quality of Life (HRQL): Daily Function, Social Participation, and Work Disruptions

Individuals with EDS/G-HSD commonly report living a “restricted life” with limitations in daily and leisure activities, social participation, and work-related activities [39,43,65]. Several daily life and work limitations are often reported by individuals with EDS/G-HSD [47]. A cross-sectional study of 466 adults with joint hypermobility syndrome observed that 52% of individuals modified their work and/or job duties as a consequence of their symptoms, and 82% of them believed that their disease impaired their work performance [11]. Another study had similar observations and found significantly lower participation in societal roles in 69 EDS/G-HSD (hEDS, classical EDS, and vascular EDS subtypes) in comparison with age- and sex-matched healthy controls. This participation included self-selected activities based on personal choices, activities leading to social appreciation, and delegated activities (Table 2). In this study, the Ghent Participation Scale scores, a measure of participation in daily and social activities, were significantly lower for hEDS/G-HSD participants (44.6 ± 9.8 vs. 55.7 ± 10.5, *p* < 0.0001) compared to healthy participants [37]. Thus, the contribution of the biopsychosocial model (Table 2) highlights the dynamic nature of reduced social participation [25,43] and the impact of social function across several health domains. This may include increased physical symptoms resulting in greater sedentary behaviours [49] and psychological sequela, such as anxiety and social isolation, which can significantly affect quality of life [47].

## 4. Discussion

Individuals with EDS/G-HSD experience a multitude of respiratory symptoms that can affect several aspects of their health. Traditionally, generic measures such as the SF-36 have been utilized to quantify HRQL; however, the literature on the impact of respiratory symptoms on HRQL has been limited in EDS/G-HSD. The biopsychosocial model was chosen to describe the relationship between respiratory symptoms and the physical and psycho-social domains of HRQL. This framework recognizes the complexity of symptoms, which is particularly important in EDS/G-HSD, where multiple symptoms can co-occur and interact with each other. However, most research studies focused on the contribution of respiratory manifestations on the physical functional domains of HRQL with the impact on the psycho-social domains not commonly evaluated.

### 4.1. Physical Domain

Physical inactivity in EDS/G-HSD is common and often attributed to musculoskeletal concerns [34,44]. Somatic symptoms, including joint pain experienced by individuals with EDS and G-HSD, may limit daily physical function and have been observed to be lower in the EDS/G-HSD population compared to healthy age- and sex-matched populations [10,66]. Decreased physical activity could be a consequence of physical impairments experienced from musculoskeletal pain, joint laxity and hypermobility, potentially restricting movement [34]. This may contribute to limited participation in social activities that are physically demanding. However, this review highlights that dyspnea and ventilatory abnormalities are also associated with reduced physical activity levels and exercise capacity. Thus, these respiratory manifestations may be an important contributor to physical functioning in EDS/G-HSD. The contribution of respiratory muscle weakness to reduced exercise capacity in EDS/G-HSD, [41] and improved outcomes following IMT is consistent with observations in other chronic conditions such as chronic obstructive pulmonary disease (COPD). IMT has been shown to improve respiratory muscle strength, symptoms, and exercise tolerance in COPD [67].

A significant consequence of reduced physical activity in EDS/G-HSD is deconditioning, which can exacerbate joint laxity, pain, and muscle weakness [64]. Given that respiratory symptoms have been associated with physical inactivity in EDS/G-HSD, they may play an important role in perpetuating the cycle of deconditioning, which can impact social participation, well-being, and HRQL [68]. Deconditioning itself can contribute to increased exertional dyspnea, as a consequence of lower anaerobic threshold and increased respiratory drive for a given amount of exertional effort [69,70]. Furthermore, individuals who experience musculoskeletal injuries tend to avoid physical activity which in turn may exacerbate deconditioning and fatigue [71]. Kinesiophobia has also been associated with fatigue severity in EDS/G-HSD [71]. It is possible that individuals with EDS/G-HSD modify their participation in physical activity to avoid the unpleasant sensation of dyspnea, which has been observed across several chronic lung conditions [72,73]. Therefore, dyspnea may be a contributing factor to kinesiophobia in EDS and G-HSD, and may accentuate the cycle of deconditioning [73].

### 4.2. Psychological and Social Domain

Respiratory symptoms may further exacerbate pain, anxiety, and avoidance of physical activity, which can result in a sedentary lifestyle and reduced social engagement. Fatigue may be influenced by factors such as dysautonomia, skeletal muscle dysfunction, and ventilatory limitations. Other determinants of fatigue in EDS/G-HSD include sleep disturbances, social functioning, and pain severity [8]. Despite the unknown mechanisms between fatigue and respiratory symptoms in EDS/G-HSD, it is often conceptualized as bidirectional in several lung conditions, and respiratory symptoms may be a key contributor of fatigue in EDS/G-HSD [74,75].

There was a high prevalence of anxiety in EDS patients with dyspnea [10]. Anxiety can aggravate dyspnea as seen in chronic lung disease populations [76,77]. Those with higher severity of respiratory symptoms have been observed to have more severe anxiety, depression, and lower HRQL. Given the overlapping and heterogeneous nature of anxiety and respiratory symptoms in EDS and G-HSD, there is a need to further understand their complex relationships to aid with clinical management. Furthermore, in other respiratory conditions such as COPD, the unpleasant sensation of dyspnea may contribute towards activity avoidance and skeletal muscle deconditioning [78,79]. Thus, given the multisystem nature of EDS/G-HSD, respiratory symptoms and its associated psychosocial influences may limit an individual’s ability to participate in social activities. This, in turn, may contribute to feelings of isolation, the loss of supportive relationships, less engagement in social activities, and reduced HRQL [43].

EDS patients often report significantly lower satisfaction with social activities compared to population normative values [80]. Individuals with EDS/G-HSD describe avoidance of social activities due to the inability to match the pace of group activities, as a consequence of physical limitations, fatigue and/or pain [65]. Since respiratory symptoms can have a negative impact on physical functioning and fatigue, they may indirectly contribute to impaired social functioning in EDS/G-HSD. Furthermore, both POTS and MCAS are potential sources of respiratory symptoms in EDS/G-HSD and these conditions have been associated with impairments across several domains of HRQL, including limitations in daily function and vocational disruptions [15,81]. Taken together, associated respiratory sequelae may disrupt daily function, family, professional and social participation, thus negatively impacting HRQL in individuals with EDS/G-HSD.

Rehabilitation programs have been shown to be an effective intervention for improving HRQL in individuals with EDS/G-HSD [82]. A systematic review by Buryk-Iggers et al. of 10 studies showed that physical and psychological benefits were observed in the EDS/G-HSD population with less than 6 weeks of multi-modal rehabilitation. With the completion of a rehabilitation program comprised of aerobic, strength training, flexibility exercises, education and cognitive-behavioral elements, clinical outcomes such as exercise capacity, muscle strength, daily function, and kinesiophobia were significantly improved [64]. These benefits with rehabilitation may be attributed to improved joint stability, reduced pain and enhanced cardiorespiratory fitness, which can modify the cycle of deconditioning [64]. Furthermore, improved cardiorespiratory fitness may alleviate some of the dyspnea experienced in the EDS/G-HSD population [83,84]. These findings may be important for health care professionals and individuals with EDS/G-HSD to help optimize physical activity levels and exercise participation.

### 4.3. Future Directions

Given the multi-systemic nature of EDS/G-HSD, identifying which symptoms may co-occur will help guide management [85]. Respiratory symptoms are often described in clusters in chronic disease states and several symptoms including fatigue, anxiety, and sleep disturbances may overlap with respiratory symptoms. Mixed-methods research approaches, utilizing both qualitative and quantitative techniques, may be useful in identifying symptom clusters in the EDS/G-HSD population. Qualitative approaches allow for open-ended and in-depth exploration of symptom experiences. This may be particularly useful in EDS/G-HSD, as many individuals report their “voices” are often not heard and their experiences not fully appreciated with currently available patient-reported outcome measures (PROMs) [86,87]. Furthermore, most studies in EDS/G-HSD evaluating respiratory symptoms have often utilized generic PROMs that assess common respiratory symptoms (i.e., dyspnea, cough, etc.); however, other manifestations such as systemic features of autonomic dysfunction or MCAS may not always be captured [13]. Thus, the open-ended nature of qualitative interviews and focus groups allows for individuals with EDS/G-HSD to voice their experiences that have not been previously evaluated with standard PROMs [88,89]. In addition, the evaluation of content, construct, and criterion validity of PROMs utilized in EDS and G-HSD patients should provide more accurate assessments [90].

There are several working theories related to the pathophysiology of dyspnea in EDS/G-HSD [12,91]. Future research that evaluates less commonly reported neurologic manifestations of disease that may contribute towards respiratory symptoms is necessitated by the presence of comorbid conditions affecting the brainstem and upper spinal cord in EDS-HSD [12,92]. Cranio-cervical instability [93] and Chiari malformations [92] may be associated with respiratory symptoms in a subset of individuals with EDS/G-HSD; however, its implications on HRQL have not been previously described. Furthermore, the present review was unable to address complex manifestations of disease linked to mechanical disturbances in EDS/G-HSD populations, such as Eagle syndrome (i.e., styloid hypertrophy) that can rarely present with airway obstruction, dysphagia, or carotid dissection [94,95]. More research is needed to address how mechanical respiratory system disruptions associated with hypermobility may affect symptoms and HRQL in EDS/G-HSD populations.

The twelve key studies included in this review had several limitations. Firstly, 50% of the studies had a cross-sectional study design limiting their inferences on relationships between respiratory symptoms and HRQL measures to a specific time period. Secondly, the majority of participants in these studies were female (87%) and had a diagnosis of hEDS or G-HSD, which limits generalizability to male individuals or those with other EDS subtypes. Finally, most studies used self-reported questionnaires with only a few studies utilizing spirometry [36,42,48], but other objective measures such as physical activity trackers were not used. Thus, future studies should aim to integrate objective respiratory measures longitudinally, and strive for a greater representation of other EDS subtypes (classical, vascular, etc.).

There is an also urgent call for the integration of patient-centered approaches in research and care for the EDS/G-HSD population [26]. Individuals with EDS/G-HSD often report difficulties in communicating their unique needs to health care providers as many providers are often not familiar with the constellation of symptoms experienced by EDS and G-HSD patients [96]. Thus, integration of individuals with EDS/G-HSD as active participants in guiding research questions through patient advisory boards and knowledge dissemination meetings is of critical importance [97]. This interaction will not only help facilitate knowledge exchange, but will help with future development of disease-specific PROMs that capture important respiratory symptoms and patient centered outcomes. Table 3 summarizes some of the knowledge gaps and future research considerations related to the management of respiratory symptoms in EDS/G-HSD.

### 4.4. Clinical Implication for Research and Practice

Individuals with EDS/G-HSD often describe having difficulties in validating their health concerns, as EDS/G-HSD is often viewed by health care providers as a musculoskeletal condition [96]. However, this narrative review provides a unique perspective on the importance of respiratory symptoms beyond musculoskeletal limitations for patients and health care providers. Given the varied constellation of respiratory symptoms and health effects on physical, psychological and social domains, individuals with EDS/G-HSD may be able to make more informed decisions regarding their daily physical and social activities. Additionally, we hope that highlighting the impact of respiratory symptoms through this review will help patients and health care providers manage these respiratory sequela [86].

The multidimensional perspective of respiratory manifestations (i.e., dyspnea, cough, autonomic dysfunction) on HRQL provides a number of research opportunities through wearable technology and electronic PROMs to further understand the trajectory and impact in individuals with EDS and G-HSD. It will also help pave the way for future behavioural interventions through physical activity counselling, exercise training, and lifestyle modifications in the setting of respiratory symptoms.

## 5. Conclusions

This review highlights that respiratory manifestations have negative effects on physical, psychological, and social domains of HRQL. Dyspnea, cough, wheezing, and expiratory flow limitations were associated with limitations in physical function and exercise intolerance in EDS and G-HSD. More research is needed to understand the specific contribution of respiratory symptoms on HRQL. In order to advance research and care in the EDS/G-HSD population, disease-specific PROMs should be developed and used both in the clinical and research settings.

## Figures and Tables

**Table 1:** Keywords used in the literature search.

Disease-Related Terms	HRQL Terms	Measure-Related Terms	Other-Related Terms
Ehlers-Danlos Syndrome OR Ehlers-Danlos OR Hypermobility Syndrome OR Hypermobility Spectrum Disorder	Quality of Life OR Activities of Daily Living OR Functional Capacity OR Social Participation	Patient-reported Outcome Measures	Lived Experience OR English Language

Keywords shown for Ovid Medline.

**Table 2:** Summary of studies related to physical, psychological, and social domains of health.

Authors and Year	Aim(s)	Study Population	Study Design/Measures	Key Findings
Jeffery (2024) [35]	To explore the voice experience, singing ability, and well-being of singers diagnosed with HSD or hEDS	N = 276 completed the survey across Europe, America, Africa, and Australia including *n* = 71 professionally trained singers, age range = 18–60, 96% female **Inclusion:** >18 years with HSD or hEDS English-speaking Singers **Exclusion:** No formal singing training	Mixed-method study Online surveys: written closed and open-ended questions Demographics Thematic descriptions	75% of singers experienced throat pain, functional voice difficulties (e.g., hoarse voice), and other laryngeal difficulties (i.e., spasms in the throat, coughing, choking) Participants experienced sudden voice breaks when singing, struggled to control their breath, pitch, intonation, and volume Comorbid conditions including dysautonomia, GERD, and POTS affect voice function (i.e., feeling faint and unable to catch one’s breath)
Reina-Gutierrez (2023) [31]	To assess functional respiratory complaints and their relationship with mental health	N = 186 hEDS from Belgium; 57% were between 36 and 55 years, 86% female **Inclusion:** >18 years with hEDS French-speaking **Exclusion:** Inability to read or comprehend questionnaires	Cross-sectional study Online surveys: emailed to participants Socio-demographics Nijmegen questionnaire—functional respiratory complaints Central sensitization inventory—central sensitivity syndromes (e.g., fibromyalgia) Brief illness perception questionnaire—cognitive representations of illness Hospital anxiety and depression scale—depression and anxiety	Cluster of people with severe functional respiratory complaints had more severe depression, anxiety, and central sensitization
Hakimi (2022) [36]	To explore lung function during exercise in hEDS patients	N = 12 hEDS from France; Mean age = 41 ± 14 years, 92% female **Inclusion:** Recent CPET Diagnosis of hEDS	Exploratory study Tests: Spirometry, incremental cardio-pulmonary exercise test, constant load exercise test Inspiratory capacity (IC) End-expiratory volume (EELV) Forced vital capacity Exercise capacity	Significant decreases in IC and increases in EELV indicative of dynamic hyperinflation Reduced exercise capacity (peak oxygen consumption)
De Baets (2022) [37]	To investigate differences in societal participation between EDS/G-HSD and healthy controls	N = 69 EDS/G-HSD (20 hEDS, 4 cEDS, 18 vEDS, 27 G-HSD), Mean age: 41 ± 17 years, 79% female **Inclusion:** Dutch or French language >16 years old Hypermobile, vascular, classical, HSD Diagnosed at Ghent University Hospital **Exclusion:** System conditions (chronic fatigue syndrome, rheumatism, diabetes, neuropathy)	Retrospective case-control study Ghent Participation Scale—Societal participation (self-performed and delegated activities)	Significantly lower societal participation in hEDS/G-HSD in comparison with healthy controls More than half of individuals changed their work and/or duties due to their symptoms
Ruiz Maya (2021) [38]	To assess the prevalence of dysautonomia, associated symptoms, and physical activity/exercise levels in EDS/G-HSD	N = 144 EDS/G-HSD; 42% hEDS 58% G-HSD Median age = 31 years, 94% female **Inclusion:** Evaluated in a cardiovascular genetics program for joint hypermobility hEDS or HSD diagnosis **Exclusion:** Alternative genetic or rheumatologic diagnosis	Retrospective chart review study Symptoms of dysautonomia Self-reported physical activity levels	Dysautonomia associated symptoms including dyspnea and chest pain were associated with lower exercise tolerance and physical activity levels
Simmonds (2019) [39]	To explore exercise perceptions, behaviours, and experiences with physiotherapy in EDS	N = 946 hEDS; 72.1% were between 18 and 40 years of age, 96% female **Inclusion:** >18 years old JHS or EDS-HT diagnosis **Exclusion:** Sticklers syndrome, Marfan syndrome, osteogenesis imperfecta or other forms of EDS	Cross-sectional study Open-ended questionnaires: Exercise barriers and thematic description of exercise beliefs	Pain, fear of injury, and fatigue were described as the most common barriers to performing exercise
Schubart (2019) [40]	To identify phenotypic EDS subgroups with distinct symptom profiles	N = 175 EDS, Median age = 42 years, 7% female **Inclusion:** Subset of patients in the HDCT cohort with EDS diagnosis Physical examination at baseline visit >21 years old	Exploratory study Data abstracted from the National Institute on Aging Intramural Research Program HRQL Symptom profile clusters	Those in the “high symptom burden” group reported the highest severity of all symptoms, the highest number of respiratory symptoms, and the lowest HRQL
Krahe (2018) [41]	To investigate predictors of fatigue and its prevalence, severity, and impact in EDS	N = 117 EDS, Mean age = 35 ± 21 years, 94% female **Inclusion:** 16–65 years old JHS or EDS-HT diagnosis **Exclusion:** Marfan syndrome Osteogenesis imperfecta EDS Type I, II, IV Stickler’s disease or Loeys-Dietz syndrome	Cross-sectional study Questionnaires: Physical Activity Index—physical activity Depression, Anxiety and Stress Scale—anxiety, stress Assessment of Quality of Life—HRQL Fatigue Severity Scale—fatigue Visual Analog Scale—orthostatic intolerance	Fatigue predictors: Joint hypermobility Orthostatic intolerance related to exercise and heat Physical activity levels Social participation Dissatisfaction with the health care system and diagnostic process Fatigue impact: Greater fatigue severity was correlated with lower HRQL and higher depression and anxiety levels
Voermans et al. (2010) [8]	To measure fatigue, its clinical relevance, and possible determinants	N = 273 from Dutch patient organization, Mean age = 40.7, 89% female **Inclusion:** >16 years old EDS diagnosis **Exclusion:** No official diagnosis of EDS Patients with incomplete responses on the CIS fatigue severity scale	Cross-sectional study Questionnaires: The Sickness Impact Profile (SIP)—functional impairment in daily life, sleep disturbances, social functioning, and social support Physical Activity The Beck Depression Inventory—psychological distress The Symptom Checklist Short Form-36 Checklist Individual Symptoms (CISs)—concentration problems Self-Efficacy Scale—fatigue Causal attribution of fatigue Visual Analog Scale—pain	77% of EDS patients suffer from severe fatigue Patients who are severely fatigued report a higher level of psychological distress Severe fatigue in EDS is related to sleep disturbances, concentration problems, social functioning, self-efficacy concerning fatigue, and pain
Morgan et al. (2007) [42]	To investigate respiratory manifestations in HSD/Benign Joint Hypermobility Syndrome (BJHS) and EDS	N = 126 BJHS N = 162 EDS Median age: 37 years, 78% female **Inclusion:** BJHS, EDS diagnosis Clinical assessment: Geographical location and ability to attend for assessment	Cross-sectional study Questionnaire: St. George’s Respiratory Questionnaire Pulmonary function tests—respiratory manifestations and lung volumes	Increased prevalence of asthma, cough, wheezing, and dyspnea with physical activity
Berglund et al. (2000) [43]	To explore the daily life experiences of people with EDS	N = 10 EDS from Sweden, Age range = 21–67 years, 63% female **Inclusion:** >18 years old Members of support group	Exploratory study Individual Interviews: Thematic description of daily life	Daily functioning: “Living restricted lives” is the main experience identified among participants describing social limitations due to physical symptoms and fears surrounding risk of injury Sleep: “Living with constant pain” contributed to trouble sleeping at night and daytime exhaustion

**Table 3:** Gaps and future directions in respiratory symptom management and care in EDS and G-HSD.

Identified Gaps in the Literature	Future Research Directions
Current research utilizes patient-reported outcome measures (PROMs) that are not disease specific and have not been validated in EDS or G-HSD.	Validation of respiratory and non-respiratory PROMs in EDS and G-HSD.
	Development of respiratory and disease-specific PROMs and instruments for using both quantitative and qualitative methodologies to ensure symptoms and outcomes are appropriately evaluated.
Lack of patient-centered approaches in guiding research studies and care.	Utilization of qualitative methodology to capture prioritized outcomes for individuals with EDS/G-HSD.
	Identify respiratory symptom management needs and priorities for advancement of patient-centered care.
	Establish patient advisory boards and opportunities for knowledge dissemination meetings.
	Identify key research questions and priorities that have a significant impact on the EDS/G-HSD population.
Respiratory symptom profile development and identification of symptom clusters given multi-morbidity.	Utilize qualitative and quantitative methods to characterize respiratory symptoms and recognition of symptom clusters.
Limited information on the mechanisms of respiratory symptoms and their relationship to key outcomes.	Investigate the pathophysiology of key respiratory symptoms (e.g., dyspnea) and assess the association of respiratory symptoms to key outcomes such as health-related quality of life.
The majority of respiratory research in EDS/G-HSD focuses on the physical domains of health.	Investigate the psychosocial domains related to respiratory manifestations in EDS/G-HSD, as part of comprehensive evaluation and symptom management.

## Data Availability

No new data was created or analyzed in this study. Data sharing is not applicable to this article.

## Supplementary Materials

The following supporting information can be downloaded at: https://www.mdpi.com/article/10.3390/jcm14124126/s1, Search Strategy Outlined.

## Acronyms

cEDS	Classical Ehlers-Danlos Syndrome
EDS	Ehlers-Danlos Syndrome
G-HSD	Generalized Hypermobility Spectrum Disorders
hEDS	Hypermobile Ehlers-Danlos Syndrome
HRQL	Health-Related Quality of Life
PROMs	Patient-Reported Outcome Measures
POTS	Postural Orthostatic Tachycardia Syndrome
MCAS	Mast Cell Activation Syndrome
