# Supplementary material for: Respiratory Manifestations and Their Physical, Psychological, and Social Impacts in Ehlers-Danlos Syndromes and Generalized Hypermobility Spectrum Disorders: A Narrative Review
Source: J Clin Med. 2025 Jun 11;14(12):4126. doi: 10.3390/jcm14124126 (PMC12194458; doi:10.3390/jcm14124126)
Supplement: Supplementary file 1 [file jcm-14-04126-s001.zip › jcm-3364548-supplementary.pdf]

Journal of
Clinical Medicine

MDPI

Supplementary Materials:

Respiratory Manifestations and their Physical, Psychological, and Social Impacts in Ehlers-Danlos Syndromes and Generalized Hypermobility Spectrum Disorders: A Narrative Review

Noor Al Kaabi [1,2,3], Encarna Camacho [1], Ani Orchanian-Cheff [4], Vanessa Silano,[1] Laura McGillis[3], Wing Ting Truong[3], W. Darlene Reid [5,6,7], Chung-Wai Chow[1,2], Clodagh M Ryan[1,2,5], Maxwell Slepian[3], Daniel Santa Mina [3,8,9] Hance Clarke[2,3,9], Nimish Mittal [2,3,8,9], Dmitry Rozenberg[1,2,3]

1 Division of Respirology, University Health Network, Toronto, ON, Canada
2 Temerty Faculty of Medicine, University of Toronto, Toronto ON, Canada
3 GoodHope Ehlers-Danlos Syndrome Program, University Health Network, Toronto, ON, Canada
4 Library and Information Services, University Health Network, Toronto, ON, Canada
5 KITE—Toronto Rehab-University Health Network, Toronto, ON, Canada
6 Department of Physical Therapy, University of Toronto, Toronto, ON, Canada
7 Interdepartmental Division of Critical Care Medicine, University of Toronto, Toronto, ON, Canada
8 Faculty of Kinesiology and Physical Education, University of Toronto, Toronto, ON, Canada
9 Department of Anesthesia and Pain Management, University Health Network, Toronto, ON, Canada

Correspondence: Dmitry Rozenberg, MD, PhD
Respirology and Lung Transplantation, Temerty Faculty of Medicine
Ehlers Danlos Syndrome Respirology Clinic
Toronto General Hospital Research Institute, University Health Network
200 Elizabeth Street, 13 EN-229 Toronto, ON, M5G 2C4, Canada
Phone: 1 416 340 4800 ext. 7358
Fax: 1 416 340 4531
Email: Dmitry.Rozenberg@uhn.ca

Table of Contents:

Search Strategy Outlined (pages 2 to 10)

Citation: To be added by editorial staff during production.

Academic Editor: Firstname Lastname

Received: date
Revised: date
Accepted: date
Published: date

Copyright: © 2025 by the authors. Submitted for possible open access publication under the terms and conditions of the Creative Commons Attribution (CC BY) license

(https://creativecommons.org/licenses/by/4.0/).

Search Methods:

A search strategy was initially developed for Ovid Medline for the concepts of Ehlers-Danlos Syndrome and quality of life, with a combination of subject headings and keywords. The search strategy was translated to other databases. Three databases were searched on January 10, 2022, April 4, 2023, and March 14, 2024: Ovid Medline, Ovid Embase, and CINAHL Complete/Ultimate (EBSCOhost). Results were limited to English language and conference materials were removed.

Database search strategies used:

Searches as Run January 10, 2022

Ovid MEDLINE(R) ALL <1946 to January 07, 2022>

Search history sorted by search number ascending

#	Searches	Results	Type
1	Ehlers-Danlos Syndrome/	3464	Advanced
2	ehlers-danlos.mp.	4516	Advanced
3	(hypermobility adj2 syndrome*).mp.	601	Advanced
4	(hypermobility adj2 spectrum adj2 disorder*).mp.	92	Advanced
5	1 or 2 or 3 or 4	4811	Advanced
6	"Quality of Life"/	230786	Advanced
7	exp patient reported outcome measures/	10603	Advanced
8	(functional adj2 capacity).mp.	21231	Advanced
9	Social Participation/	3041	Advanced
10	exp "Activities of Daily Living"/	112724	Advanced
11	(quality adj2 life).mp.	400365	Advanced
12	(lived adj2 experience*).mp.	8703	Advanced
13	or/6-12	521650	Advanced
14	5 and 13	157	Advanced
15	limit 14 to English language	151	Advanced
16	remove duplicates from 15	150	Advanced

Embase <1974 to 2022 January 07>

Search history sorted by search number ascending

#	Searches	Results	Type
1	Ehlers Danlos syndrome/	5636	Advanced
2	(hypermobility adj2 syndrome*).mp.	983	Advanced
3	(hypermobility adj2 spectrum adj2 disorder*).mp.	123	Advanced
4	1 or 2 or 3	6252	Advanced
5	exp "quality of life"/	561604	Advanced
6	patient-reported outcome/	36096	Advanced
7	daily life activity/	100382	Advanced

8	functional status/	59458	Advanced
9	social participation/	7910	Advanced
10	personal experience/	49679	Advanced
11	or/5-10	758643	Advanced
12	4 and 11	339	Advanced
13	limit 12 to (conference abstract or conference paper or "conference review" or conference proceeding)	118	Advanced
14	12 not 13	221	Advanced
15	remove duplicates from 14	220	Advanced
16	limit 15 to English language	211	Advanced

#	Query	Limiters/Expanders	Last Run Via	Results
S13	S4 AND S11	Expanders - Apply equivalent subjects Narrow by Language: - English Search modes - Boolean/Phrase	Interface - EBSCOhost Research Databases Search Screen - Advanced Search Database - CINAHL Complete	60
S12	S4 AND S11	Expanders - Apply equivalent subjects Search modes - Boolean/Phrase	Interface - EBSCOhost Research Databases Search Screen - Advanced Search Database - CINAHL Complete	60
S11	S5 OR S6 OR S7 OR S8 OR S9 OR S10	Expanders - Apply equivalent subjects Search modes - Boolean/Phrase	Interface - EBSCOhost Research Databases Search Screen - Advanced Search Database - CINAHL Complete	270,932
S10	(MH "Life Experiences+")	Expanders - Apply equivalent subjects Search modes - Boolean/Phrase	Interface - EBSCOhost Research Databases Search Screen - Advanced Search Database - CINAHL Complete	48,550
S9	(MH "Social Participation")	Expanders - Apply equivalent subjects Search modes - Boolean/Phrase	Interface - EBSCOhost Research Databases Search Screen - Advanced Search Database - CINAHL Complete	5,636
S8	(MH "Functional Status")	Expanders - Apply equivalent subjects Search modes - Boolean/Phrase	Interface - EBSCOhost Research Databases Search Screen - Advanced Search Database - CINAHL Complete	26,362
S7	(MH "Activities of Daily Living+")	Expanders - Apply equivalent subjects Search modes - Boolean/Phrase	Interface - EBSCOhost Research Databases Search Screen - Advanced Search	76,917

			Database - CINAHL Complete	
S6	(MH "Patient-Reported Outcomes")	Expanders - Apply equivalent subjects Search modes - Boolean/Phrase	Interface - EBSCOhost Research Databases Search Screen - Advanced Search Database - CINAHL Complete	3,946
S5	(MH "Quality of Life+")	Expanders - Apply equivalent subjects Search modes - Boolean/Phrase	Interface - EBSCOhost Research Databases Search Screen - Advanced Search Database - CINAHL Complete	135,360
S4	S1 OR S2 OR S3	Expanders - Apply equivalent subjects Search modes - Boolean/Phrase	Interface - EBSCOhost Research Databases Search Screen - Advanced Search Database - CINAHL Complete	852
S3	TI hypermobility N2 spectrum N2 disorder* OR AB hypermobility N2 spectrum N2 disorder*	Expanders - Apply equivalent subjects Search modes - Boolean/Phrase	Interface - EBSCOhost Research Databases Search Screen - Advanced Search Database - CINAHL Complete	23
S2	TI hypermobility N2 syndrome* OR AB hypermobility N2 syndrome*	Expanders - Apply equivalent subjects Search modes - Boolean/Phrase	Interface - EBSCOhost Research Databases Search Screen - Advanced Search Database - CINAHL Complete	281
S1	(MH "Ehlers-Danlos Syndrome")	Expanders - Apply equivalent subjects Search modes - Boolean/Phrase	Interface - EBSCOhost Research Databases Search Screen - Advanced Search Database - CINAHL Complete	671

Searches as Run April 4, 2023

Ovid MEDLINE(R) ALL <1946 to April 03, 2023>

#	Searches	Results	Type
1	Ehlers-Danlos Syndrome/	3680	Advanced
2	ehlers-danlos.mp.	4854	Advanced
3	(hypermobility adj2 syndrome*).mp.	643	Advanced
4	(hypermobility adj2 spectrum adj2 disorder*).mp.	136	Advanced
5	1 or 2 or 3 or 4	5168	Advanced
6	"Quality of Life"/	263029	Advanced
7	exp patient reported outcome measures/	13263	Advanced
8	(functional adj2 capacity).mp.	22737	Advanced
9	Social Participation/	3387	Advanced
10	exp "Activities of Daily Living"/	119177	Advanced
11	(quality adj2 life).mp.	445900	Advanced
12	(lived adj2 experience*).mp.	11446	Advanced
13	or/6-12	577552	Advanced
14	5 and 13	191	Advanced
15	limit 14 to English language	185	Advanced
16	remove duplicates from 15	184	Advanced
17	limit 16 to yr="2022 -Current"	33	Advanced

Embase <1974 to 2023 April 03>

#	Searches	Results	Type
1	Ehlers Danlos syndrome/	6315	Advanced
2	(hypermobility adj2 syndrome*).mp.	1064	Advanced
3	(hypermobility adj2 spectrum adj2 disorder*).mp.	192	Advanced
4	1 or 2 or 3	6984	Advanced
5	exp "quality of life"/	638687	Advanced
6	patient-reported outcome/	51408	Advanced
7	daily life activity/	112111	Advanced
8	functional status/	68801	Advanced
9	social participation/	9645	Advanced
10	personal experience/	60513	Advanced
11	or/5-10	872137	Advanced
12	4 and 11	431	Advanced
13	limit 12 to (conference abstract or conference paper or "conference review" or conference proceeding)	136	Advanced
14	12 not 13	295	Advanced
15	remove duplicates from 14	292	Advanced
16	limit 15 to English language	282	Advanced
17	limit 16 to yr="2022 -Current"	62	Advanced

#	Query	Limiters/Expanders	Last Run Via	Results

S14	S4 AND S11	Limiters - Published Date: 20220101-20231231<br>Expanders - Apply equivalent subjects<br>Search modes - Boolean/Phrase	Interface - EBSCOhost Research Databases<br>Search Screen - Advanced Search<br>Database - CINAHL Ultimate	15
S13	S4 AND S11	Expanders - Apply equivalent subjects<br>Search modes - Boolean/Phrase	Interface - EBSCOhost Research Databases<br>Search Screen - Advanced Search<br>Database - CINAHL Ultimate	75
S12	S4 AND S11	Expanders - Apply equivalent subjects<br>Search modes - Boolean/Phrase	Interface - EBSCOhost Research Databases<br>Search Screen - Advanced Search<br>Database - CINAHL Ultimate	75
S11	S5 OR S6 OR S7 OR S8 OR S9 OR S10	Expanders - Apply equivalent subjects<br>Search modes - Boolean/Phrase	Interface - EBSCOhost Research Databases<br>Search Screen - Advanced Search<br>Database - CINAHL Ultimate	302,546
S10	(MH "Life Experiences+")	Expanders - Apply equivalent subjects<br>Search modes - Boolean/Phrase	Interface - EBSCOhost Research Databases<br>Search Screen - Advanced Search<br>Database - CINAHL Ultimate	57,863
S9	(MH "Social Participation")	Expanders - Apply equivalent subjects<br>Search modes - Boolean/Phrase	Interface - EBSCOhost Research Databases<br>Search Screen - Advanced Search<br>Database - CINAHL Ultimate	6,672
S8	(MH "Functional Status")	Expanders - Apply equivalent subjects<br>Search modes - Boolean/Phrase	Interface - EBSCOhost Research Databases<br>Search Screen - Advanced Search<br>Database - CINAHL Ultimate	29,674
S7	(MH "Activities of Daily Living+")	Expanders - Apply equivalent subjects<br>Search modes - Boolean/Phrase	Interface - EBSCOhost Research Databases<br>Search Screen - Advanced Search	82,588

			Database - CINAHL Ultimate	
S6	(MH "Patient-Reported Outcomes")	Expanders - Apply equivalent subjects<br>Search modes - Boolean/Phrase	Interface - EBSCOhost Research Databases<br>Search Screen - Advanced Search<br>Database - CINAHL Ultimate	5,595
S5	(MH "Quality of Life+")	Expanders - Apply equivalent subjects<br>Search modes - Boolean/Phrase	Interface - EBSCOhost Research Databases<br>Search Screen - Advanced Search<br>Database - CINAHL Ultimate	149,896
S4	S1 OR S2 OR S3	Expanders - Apply equivalent subjects<br>Search modes - Boolean/Phrase	Interface - EBSCOhost Research Databases<br>Search Screen - Advanced Search<br>Database - CINAHL Ultimate	926
S3	TI hypermobility N2 spectrum N2 disorder* OR AB hypermobility N2 spectrum N2 disorder*	Expanders - Apply equivalent subjects<br>Search modes - Boolean/Phrase	Interface - EBSCOhost Research Databases<br>Search Screen - Advanced Search<br>Database - CINAHL Ultimate	39
S2	TI hypermobility N2 syndrome* OR AB hypermobility N2 syndrome*	Expanders - Apply equivalent subjects<br>Search modes - Boolean/Phrase	Interface - EBSCOhost Research Databases<br>Search Screen - Advanced Search<br>Database - CINAHL Ultimate	293
S1	(MH "Ehlers-Danlos Syndrome")	Expanders - Apply equivalent subjects<br>Search modes - Boolean/Phrase	Interface - EBSCOhost Research Databases<br>Search Screen - Advanced Search<br>Database - CINAHL Ultimate	736

Searches as run March 14, 2024

Ovid MEDLINE(R) ALL <1946 to March 13, 2024>

#	Searches	Results	Type

1	Ehlers-Danlos Syndrome/	3791	Advanced
2	ehlers-danlos.mp.	5109	Advanced
3	(hypermobility adj2 syndrome*).mp.	679	Advanced
4	(hypermobility adj2 spectrum adj2 disorder*).mp.	183	Advanced
5	1 or 2 or 3 or 4	5437	Advanced
6	"Quality of Life"/	284118	Advanced
7	exp patient reported outcome measures/	14786	Advanced
8	(functional adj2 capacity).mp.	23852	Advanced
9	Social Participation/	3595	Advanced
10	exp "Activities of Daily Living"/	122950	Advanced
11	(quality adj2 life).mp.	481609	Advanced
12	(lived adj2 experience*).mp.	13768	Advanced
13	or/6-12	620058	Advanced
14	5 and 13	220	Advanced
15	limit 14 to English language	213	Advanced
16	remove duplicates from 15	213	Advanced
17	limit 16 to yr="2023 -Current"	44	Advanced

Embase <1974 to 2024 March 13>

#	Searches	Results	Type
1	Ehlers Danlos syndrome/	6630	Advanced
2	(hypermobility adj2 syndrome*).mp.	1102	Advanced
3	(hypermobility adj2 spectrum adj2 disorder*).mp.	234	Advanced
4	1 or 2 or 3	7332	Advanced
5	exp "quality of life"/	683753	Advanced
6	patient-reported outcome/	59353	Advanced
7	daily life activity/	119283	Advanced
8	functional status/	74152	Advanced
9	social participation/	10630	Advanced
10	personal experience/	67847	Advanced
11	or/5-10	938337	Advanced
12	4 and 11	485	Advanced
13	limit 12 to (conference abstract or conference paper or "conference review" or conference proceeding)	147	Advanced
14	12 not 13	338	Advanced
15	remove duplicates from 14	338	Advanced
16	limit 15 to English language	327	Advanced
17	limit 16 to yr="2023 -Current"	59	Advanced

#	Query	Limiters/Expanders	Last Run Via	Results

S1	(MH "Ehlers-Danlos Syndrome")	Expanders - Apply equivalent subjects<br>Search modes - Boolean/Phrase	Interface - EBSCOhost Research Databases<br>Search Screen - Advanced Search<br>Database - CINAHL Ultimate	771
S2	TI hypermobility N2 syndrome* OR AB hypermobility N2 syndrome*	Expanders - Apply equivalent subjects<br>Search modes - Boolean/Phrase	Interface - EBSCOhost Research Databases<br>Search Screen - Advanced Search<br>Database - CINAHL Ultimate	299
S3	TI hypermobility N2 spectrum N2 disorder* OR AB hypermobility N2 spectrum N2 disorder*	Expanders - Apply equivalent subjects<br>Search modes - Boolean/Phrase	Interface - EBSCOhost Research Databases<br>Search Screen - Advanced Search<br>Database - CINAHL Ultimate	56
S4	S1 OR S2 OR S3	Expanders - Apply equivalent subjects<br>Search modes - Boolean/Phrase	Interface - EBSCOhost Research Databases<br>Search Screen - Advanced Search<br>Database - CINAHL Ultimate	966
S5	(MH "Quality of Life+")	Expanders - Apply equivalent subjects<br>Search modes - Boolean/Phrase	Interface - EBSCOhost Research Databases<br>Search Screen - Advanced Search<br>Database - CINAHL Ultimate	157,039
S6	(MH "Patient-Reported Outcomes")	Expanders - Apply equivalent subjects<br>Search modes - Boolean/Phrase	Interface - EBSCOhost Research Databases<br>Search Screen - Advanced Search<br>Database - CINAHL Ultimate	7,455
S7	(MH "Activities of Daily Living+")	Expanders - Apply equivalent subjects<br>Search modes - Boolean/Phrase	Interface - EBSCOhost Research Databases<br>Search Screen - Advanced Search<br>Database - CINAHL Ultimate	81,377
S8	(MH "Functional Status")	Expanders - Apply equivalent subjects<br>Search modes - Boolean/Phrase	Interface - EBSCOhost Research Databases<br>Search Screen - Advanced Search	31,739

			Database - CINAHL Ultimate	
S9	(MH "Social Participation")	Expanders - Apply equivalent subjects<br>Search modes - Boolean/Phrase	Interface - EBSCOhost Research Databases<br>Search Screen - Advanced Search<br>Database - CINAHL Ultimate	7,332
S10	(MH "Life Experiences+")	Expanders - Apply equivalent subjects<br>Search modes - Boolean/Phrase	Interface - EBSCOhost Research Databases<br>Search Screen - Advanced Search<br>Database - CINAHL Ultimate	64,221
S11	S5 OR S6 OR S7 OR S8 OR S9 OR S10	Expanders - Apply equivalent subjects<br>Search modes - Boolean/Phrase	Interface - EBSCOhost Research Databases<br>Search Screen - Advanced Search<br>Database - CINAHL Ultimate	316,415
S12	S4 AND S11	Expanders - Apply equivalent subjects<br>Search modes - Boolean/Phrase	Interface - EBSCOhost Research Databases<br>Search Screen - Advanced Search<br>Database - CINAHL Ultimate	94
S13	S4 AND S11	Limiters - Publication Date: 20230101-20241231<br>Expanders - Apply equivalent subjects<br>Search modes - Boolean/Phrase	Interface - EBSCOhost Research Databases<br>Search Screen - Advanced Search<br>Database - CINAHL Ultimate	23

Disclaimer/Publisher's Note: The statements, opinions and data contained in all publications are solely those of the individual author(s) and contributor(s) and not of MDPI and/or the editor(s). MDPI and/or the editor(s) disclaim responsibility for any injury to people or property resulting from any ideas, methods, instructions or products referred to in the content.
